# Monthly Summary

**Published:** 1895-02

**Authors:** 


### Monthly Summary.

FAILURES TO BE AVOIDED.—1. Neglect to remove calculus and to properly care for the gums. Many when they attempt to do this fail to do it thoroughly; fail to remove all the deposits beneath the gums. Frequently they overlook deposits and pockets on the proximate surfaces of the teeth.

2. Neglect to study the lines of occlusion before building up to restore the lost portion of a tooth, so that it is built too high or not high enough, or not in proper form.

3. Occluso-proximate fillings are subject to greater strain and require firmer anchorage where situated upon the mesial side of lower teeth, and distal side of the lower or mesial of the upper. This because of natural occlusion and backward movement of the lower or the upper in mastication.

4. Neglect to keep proximate fillings dry, with rubber-dam in place till well polished and closely examined.

5. The long-continued pressure of packing gold to make a large filling drives the blood somewhat from the peridental membrane, and the root of the tooth is driven more closely into its alveolar cell. The filling may be ground so as to just escape occluding force, or it may just touch an opposing cusp. Reaction setting in, the peridental blood supply is restored, perhaps increased—consequence, the tooth is elongated (apparently) the filling "strikes" and the patient may have a "sore" tooth, or the safety of the filling may be jeopardized.

6. Neglect to grind off fillings on the occlusal surfaces from time to time to correspond to the wearing away of the adjoining tooth substance.

7. Many fillings are closely adapted at the margins when finished, but as they are worn or ground down in course of time, they leak because they are not closely packed against the walls all the way up.—*Dr. Townsend in Items of Interest.*

---

FILLING ROOTS.—In Dr. Frank Abbott's method of filling root canals he very seldom uses arsenic to devitalize a tooth pulp, and when he does it is only to relieve pain. For a tooth with the remains of a dead pulp, he opens into the pulp chamber so that he can have easy access to the canals. He then uses a one in ten thousand solution of the bichlorid of mercury (one grain to twenty ounces), syringing all the canals as thoroughly as possible. With a broach he probes into the canal so as to stir up the contents, and syringes again, repeating the process till the canals are clean, and the solution in coming out will not stain a white napkin. When he has thoroughly washed it he fills it with oxichloride of zinc, to which he adds one drop of a one in two thousand bichlorid solution. This so mummifies or holds the substance which remains in the root as to give no trouble. He cleans out the canals and fills at the same sitting before dismissing the patient, painting over the gum with aconit and iodin, as a counterirritant.

Dr. Emil Schreier prepares a mixture of metallic sodium and potassium (about two parts sodium and one part potassium) of such a consistency that when a platina broach is plunged into it, through the parrafin coating a film of the alloy will adhere and be carried on the broach, and the broach thus laden is passed into the moist canal. The metal decomposes the watery contents, liberating the hydrogen and forming the hydroides of the metals (caustic potash and caustic soda), which in the nascent state actively decompose the organic matter of the pulp, saponifying it, so that it is readily washed out with water. This leaves the canal very clean and sweet.

The substance destroys the germs present partly by the heat evolved and partly by the chemical products. Of course this method is only applied with the rubber-dam in position to protect adjacent tissues.—*Review.*

---

TIN AND AMALGAM FILLING.—Some years ago I wrote an article on the use of amalgam, which was criticised rather severely; and by some I was called a "gold crank." Since that time, as before, I have been keeping a record of all my work; and to-day stand firmer than ever by my position. Though I may be a gold crank, I know that all cements will dissolve in the mouth under ordinary circumstances (in spite of the label on the bottle being "insoluble"); and I do not believe in crowning teeth when they can be properly filled. I never fill teeth with amalgam when I can get my patients to understand that gold, tin or gutta-percha possesses better tooth saving properties. In fact, I have never seen an amalgam that will save teeth under ordinary circumstances. By cutting through from the grinding surface, forming a compound cavity, and filling at least half full with tin, and finishing with amalgam, I can get tolerably good results. The object in filling teeth is not to have the fillings "stay in," but to preserve the teeth for as long a period of usefulness as possible, and a tin filling covered with amalgam gives a combination of tooth preserving property and hardness of surface for grinding purposes that cannot be had in either used separately.—*G. Chisholm*, *in Items of Interest.*

---

MAKING STEEL CROWN DIES.—The making of steel crown dies and the use of a block of wood as a counter die has not, to my knowledge, appeared in any of the dental journals.

To make a steel die that will never wear out, procure a piece of round stuff, half-inch for molars, three-eighths for bicuspids. Take them to a blacksmith and have them cut into as many three-inch pieces as you want dies. Each piece is then heated to redness and one end driven into the crown die-plate, which will give a steel cameo of the cusps and sulci complete. The dies are afterward placed in a vise and filed to any shape or size desired, being careful not to get the neck smaller than the crown.

For either the steel or fusible metal dies, a block of close-grained pine or spruce is all that is necessary for a counter. By driving the die into the end of the block you have as fine a counter as one could wish. A little oil placed in the counter will prevent crown from sticking.

A draw-plate is very handy with these dies, but is not necessary, as a piece of plate may be drawn down into shape over a series of hard wood mandrels, driven carefully into different sized holes in the block.—FRANK B. NORRIS, in *Items of Interest.*

---

BLEACHING TEETH.—Saturate the dentine with strong sodium peroxide, followed by treatment with dilute hydrochloric acid, to neutralize the alkali. Wash with hot water.—*E. C. Kirk*, *in Items of Interest.*

COAGULANTS.—Since Dr. A. W. Harlan, of Chicago, in 1889, asserted that coagulants, such as carbolic acid and bichloride of mercury, should not be used in root-canals when antisepsis is desired, there has been much controversy upon the subject. Dr. Harlan has reiterated his opinion as here given in the *Dental Review*, and upon various other occasions, and many practitioners now agree with him and have laid aside the coagulant medicines when disinfection is required in pulpless teeth.

Dr. Edward C Kirk, in *Cosmos*, combats the statements of Dr. Harlan upon this subject, and argues from a long line of experiments that coagulants are exactly the medicines that should be used. Dr. James Truman supports Dr. Kirk, and in an exhaustive article before the Academy of Stomatology in Philadelphia, December 10, 1894, detailing laborious tests upon the penetrating power of coagulants, and among other conclusions, says that "in proportion to the coagulating power of the agent will be its penetrating force independent of gravitation," and that carbolic acid, zinc chloride, etc., penetrated albumen freely. In the discussion which followed the reading of Professor Truman's paper, Dr. Harlan happened to be present and denied that carbolic acid, creosote, bichloride of mercury and zinc chloride were powerful penetrators of albuminous matter in living teeth. He announced that he would read a paper upon the subject before the January meeting of the First District Dental Society of New York, and until that time did not care to give his reasons in full. The profession will look with interest to the further elucidation of the subject.— *Western Dental Journal.*

---

SAWDUST BREAD.—Leon Lilienfeld, a young chemist and assistant of Prof. Kossel, has made a discovery which scientists here deem of great importance for the future, though in itself it is, perhaps, not of great moment. He has succeeded in preparing artificially a chemical product which possesses all the properties of soluble peptones, including those of easy digestibility. Werner von Siemens, it was who, in 1886, prophesied that chemistry by-and-by would be able to prepare, out of waste material in nature, food stuffs, suited to the human palate and stomach. This discovery by young Lilienfeld is looked on here as the first step in that direction. The second one perhaps, is the invention of "wood bread," more correctly speaking, sawdust bread, which is now being baked in a Berlin establishment at the rate of two hundred-weight a day. The mixture is two-thirds to three-fourths sawdust, and one-third or one-fourth rye flour. By a chemical process the sawdust loses its texture and taste, and liberates its saccharine and nutritive elements, which, in combination with the rye flour, are baked into biscuits and bread. The price of this bread is five marks ($1.25) per hundred weight. Thus far it has been used solely as food for the horses of the large Berlin horse-car company, one horse disposing of from twenty to thirty pounds of this delicacy a day.—*Evening Post.*

---

TEETHING POWDERS.—It takes eighteen and one-ninth grains to the ounce of water to make a four per cent. solution.

I have always found the following an excellent teething powder.

| | | |
|---|---|---|
| R. | Hydrarg. Chlor. Mite - - - - | 1 grain. |
| | Pulvis Opii. - - - - - - - | 1 grain. |
| | Bismuth Subnit. - - - - - - | 30 grains. |
| | Pulv. Cinnamomi - - - - | 30 grains. |

M. et ft. powders No. 12.

Sig: One night and morning if necessary.

J. C. LEAKE, M. D., in *Medical Brief.*

---

COFFEE KILLS GERMS—Coffee has been found by a German investigator to possess marked germicial properties. Pure coffee, of the ordinary strength in which it is utilized as a beverage, killed cholera bacilli in three hours, and typhus bacilli in twenty-four hours. The anti-bacterial substances seem to be developed in the coffee bean by the roasting process.—*Literary Digest.*

THE PARTS THAT DO NOT GROW OLD.—In his work on the senile heart Dr. Balfour tells us that there are two parts of the human organism which, if wisely used, "largely escape senile failure." These two are the brain and the heart. Persons who think have often wondered why brain workers, great statesmen, and others, should continue to work with almost unimpaired mental activity and energy up to a period when most of the organs and functions of the body are in a condition of advanced senile decay. There is a physiological reasons for this, and Dr. Balfour tells us what it is. The normal brain, he affirms, "remains vigorous to the last," and that "because its nutrition is specially provided for." About middle life or a little later, the general arteries of the body begin to lose their elasticity and to slowly but surely dilate. They become, therefore, much less efficient carriers of the nutrient blood to the capillary areas. But this is not the case with the internal carotids, which supply the capillary areas of the brain. On the contrary, those large vessels "continue to retain their pristine elasticity, so that the blood-pressure remains normally higher than within the capillary area of any other organ in the body. The cerebral blood-paths being thus kept open, the brain tissue is kept better nourished than the other tissues of the body." Who is there among those who have reached or passed middle age that will not be rejoiced to find such admirable physiological warrant for the belief that the brain may continue to work, and even to improve, almost to the very last hour of life?—*Medical Record.*

---

THE EFFECTS OF INTENSE COLD UPON THE MIND.—Extreme cold, as is well known, exerts a benumbing influence upon the mental faculties. Almost everyone who has been exposed, for a longer or shorter period, to a very low temperature has noted a diminution in will-power, and often a temporary weakening of the memory. Perhaps the largest scale upon which this action has ever been studied was during the retreat of the French from Moscow. The troops suffered extremely from hunger, fatigue, and cold—from the latter perhaps most of all. A German physician who accompanied a detachment of his countrymen, has left an interesting account of their trials during this retreat. From an abstract of this paper by Dr. Rose, in the *New Yorker Medicinische Monatschrift,* we find that of the earliest symptoms referable to the cold was a loss of memory. This was noted in the strong as well as those who were already suffering from the effects of the hardships to which they had been exposed. With the first appearance of a moderately low temperature (about five degrees above zero Fahrenheit), many of the soldiers were found to have forgotten the names of the most ordinary things about them, as well as those of the articles of food for the want of which they were perishing. Many forgot their own names and those of their comrades. Others showed more pronounced symptoms of mental disturbance, and not a few became incurably insane, the type of their insanity resembling very closely senile dementia. The cold was probably not alone responsible for these effects, for a zero temperature is rather stimulating than paralyzing in its action upon the well fed and the healthy. These men were half-starved, poorly clad, worn out with long marching, many already weakened by dysentery and other diseases, and all mentally depressed; as an army in defeat always is. It needed, therefore, no very unusual degree of cold to produce the psychic effects observed under other circumstances only as a consequence of exposure to an extreme low temperature. —*Medical Record.*

---

DRY SURGERY IN GERMANY.—The American practitioners and students of medicine who have been trained to look upon irrigation as essential to the aseptic handling of wounds in their after-treatment, are always quite astonished at the apparent disregard the German surgeons seem to have for this method of securing good results. While in Gottingen, I was present every day at the surgical policlinic held daily by Professor Rosenbach, and I do not believe I saw a drop of water or other irrigation fluid used during the whole time. The patient is brought in, the dressings removed, the wound examined, squeezed lightly, oozing pus, is wiped off and dressings, dry or wet as may be necessary, are reapplied. Even in the treatment of deep abscesses, or where neurotic processes are going on, irrigation is never resorted to the surgeon seeming to have all faith in his drainage tubes without resorting to the stream of bichloride water as used by our American surgeons. They probably get just as good results here in Germany as we do in the United States, but their methods of wound-handling are certainly not so cleanly as those used in the latter country.—Berlin Correspondent of the *Memphis Medical Monthly.*

---

PAINLESS EXTRACTION THIRTY YEARS AGO.—"Doctor, I understand that you pull teeth without pain, by mesmerism. Is it so?"

It was news to me, but I took in the situation on the instant, and answered: "Yes, madam, that is what I do."

"Now, doctor, is this true, or are you humbugging people?"

"Why, madam, do I look like a humbug; haven't you heard that it is true."

"Yes, I have, and I've come to see."

I told her to look into my eye without blinking till hers closed, and she would not know when the teeth came out. She obeyed orders, and declared she never felt the pain of extracting.—*J. W. Greene in Items of Interest.*

---

FAST LIVING.—The most remarkable instance of rapid growth is said to be recorded by the French Academy in 1729. It was a boy six years of age, five feet six inches in height. At the age of five his voice changed, at six his beard had grown, and he appeared a man of thirty. He possessed great physical strength, and could easily lift to his shoulders and carry bags of grain weighing two hundred pounds. His decline was as rapid as his growth. At eight his hair and beard were gray; at ten he tottered in his walk, his teeth fell out, and his hands became palsied; at twelve he died with every outward sign of extreme old age.—*Times and Register.*

WHAT HOT SPRINGS AND HOT BATHS WILL DO.—The United States Government has established a hospital at Hot Springs, Ark., for the treatment of soldiers and officers of the army. The hospital is now under charge of Surgeon Alfred A. Woodhull, who issues through the adjutant-general's office this circular: "Relief may reasonably be expected at the Hot Springs in the following conditions: In the various forms of gout and rheumatism, after the acute or inflammatory stage, neuralgia, especially when depending upon gout, rheumatism, metallic or malarial poisoning; paralysis, not of organic origin; the earlier stages of locomotor ataxia, or tabes; the early stages, only, of Bright's disease, diseases of the urinary organs; functional diseases of the liver; gastric dyspepsia not of organic origin; chronic diarrhœa; catarrhal affections of the digestive and respiratory tracts; chronic skin diseases, especially the squamous varieties; and chronic conditions due to malarial infection. . . . The Hot Springs water is contraindicated in all acute inflammatory diseases, tuberculosis, organic disease of the heart or brain; cancer and other malignant disease, aneurism, and all cases where stimulation of the circulation is to be avoided."

This seems to be a temperate statement of what hot water copiously taken, combined with proper hygienic surroundings, will do at Hot Springs, and we would add, at any place where pure water is supplied in abundance and at various temperatures.—*Medical Record.*

---

A SURVIVAL.—In Vienna an old man named Karl Edelmoser, the proprietor of a shaving saloon and well-known throughout the whole district also as a tooth-operator, has had to appear before a criminal court to answer a charge of malpractice. The indictment urged that Edelmoser had for many years been in the habit of extracting teeth on a very extensive scale. The fee appears to have been 30 kreuzers (about 25 cents.) Edelmoser explained to the judge that he felt justified in extracting teeth, as he possessed a surgical diploma, which he then produced. The judge explained that the yellow document had been obsolete for some time in consequence of later legislation, and the defendant must have been well aware of this. The operator further urged that he had been in the employ of a certificated dentist for twenty-three years and thus attained great skill, but the judge held this did not quality him to practice dentistry. The complaint of the police district dentist laid against the defendant was that he had extracted a decayed tooth with a dirty instrument for a cook, and this resulted in periostitis. The judge ordered Edelmoser to pay a fine of 15 gulden (30 shillings), or, in default, three days' imprisonment, and the same sum as compensation to the patient.—*British Journal of Dental Science.*

---

DEATH IS PAINLESS.—Dr. Cyrus Edson, in *North American Review*, says: "Nothing is more common than to hear from the pulpit pictures in words of excitement, of alarm, of terror, of the death-beds of those who have not lived religious lives; yet, as a rule, if these pictures are supposed to be those of the unfortunates at the moment of death, they are utterly false. In point of fact, ninety-nine of every hundred human beings are unconscious for several hours before death comes to them. All the majesty of intellect, the tender beauty of thought, of sympathy, or charity, the very love of those for whom love has filled all waking thoughts, disappear. As a little baby just born into the world is but a little animal, so the sage, the philosopher, the hero, the statesman, he whose thoughts or deeds have writ themselves large in the history of the world, becomes but a dying animal at the last. A merciful unconsciousness sets in, as the mysterious force we call life slowly takes leave of its last citadel, the heart, and what is has become what was. This is death."

